# Contribution of Flavonoids and Iridoids to the Hypoglycaemic, Antioxidant, and Nitric Oxide (NO) Inhibitory Activities of *Arbutus unedo* L.

**DOI:** 10.3390/antiox9020184

**Published:** 2020-02-22

**Authors:** Maria Concetta Tenuta, Brigitte Deguin, Monica Rosa Loizzo, Annabelle Dugay, Rosaria Acquaviva, Giuseppe Antonio Malfa, Marco Bonesi, Chouaha Bouzidi, Rosa Tundis

**Affiliations:** 1 Department of Pharmacy, Health and Nutritional Sciences, University of Calabria, 87036 Rende (Cosenza), Italy; mary.tn2006@hotmail.it (M.C.T.); monica_rosa.loizzo@unical.it (M.R.L.); marco.bonesi@unical.it (M.B.); rosa.tundis@unical.it (R.T.); 2 Université de Paris, UFR de Pharmacie de Paris, U.M.R. n°8038, -CiTCoM- (CNRS, Université de Paris), F-75006 Paris, France; annabelle.dugay@parisdescartes.fr (A.D.); chouaha.bouzidi@parisdescartes.fr (C.B.); 3 Department of Drug Science - Biochemistry Section, University of Catania, Viale A. Doria 6, 95125 Catania, Italy; racquavi@unict.it (R.A.); g.malfa@unict.it (G.A.M.)

**Keywords:** strawberry tree, extraction procedures, chemical profiles, metabolic diseases, functional products

## Abstract

This study aims at investigating the contribution of two classes of compounds, flavonoids and iridoids, to the bioactivity of *Arbutus unedo* L. leaves and fruits. The impact of different extraction procedures on phytochemicals content and hypoglycemic, antioxidant, and nitric oxide (NO) inhibitory activities of *A. unedo* fresh and dried plant materials was investigated. Ellagic acid 4-*O*-β-D-glucopyranoside, kaempferol 3-*O*-glucoside, and norbergenin were identified for the first time in this genus by using liquid chromatography-electrospray ionization-quadrupole-time of flight-mass spectrometry (LC-ESI-QTOF-MS). Three iridoids (gardenoside, geniposide, unedoside) are specifically identified in the leaves. Interestingly, asperuloside was extracted only from dried fruits by ethanol with Soxhlet apparatus. Extracts were screened for their potential antioxidant activities by using the 2,2′-azino-bis(3-ethylbenzothiazoline-6-sulfonic acid) (ABTS), 2,2-diphenyl-1-picrylhydrazyl (DPPH), Ferric Reducing Activity Power (FRAP), and β-carotene bleaching tests. Based on the Global Antioxidant Score (GAS) calculation, the most promising antioxidant extract was obtained by hydroalcoholic maceration of dried leaves that showed half maximal inhibitory concentration (IC_50_) of 0.42 and 0.98 μg/mL in ABTS and DPPH assays, respectively. The hypoglycaemic activity was investigated by α-amylase and α-glucosidase inhibition tests. Extracts obtained by ethanol ultrasound extraction of fresh leaves and hydroalcoholic maceration of fresh fruits (IC_50_ of 19.56 and 28.42 μg/mL, respectively) are more active against α-glucosidase than the positive control acarbose (IC_50_ of 35.50 μg/mL). Fruit extracts exhibited the highest anti-inflammatory activity.

## 1. Introduction

Degenerative diseases occur because of the continuous deterioration of cells and tissues that ultimately affects the major organs. Both oxidative stress and inflammation are considered major role players in the pathogenesis of chronic degenerative diseases including cardiovascular diseases, rheumatoid arthritis, and diabetes mellitus [1]. At present, although several synthetic drugs are used to attenuate oxidative stress and inflammation-mediated degenerative diseases, none is free from side effects. Over the past decades, experimental progresses have been made in the use of natural products against different chronic degenerative diseases mainly targeting oxidative stress and inflammation, which are the major culprits in the pathogenesis of these diseases with high social and economic impact [2]. Numerous studies have demonstrated that natural compounds are important therapeutic agents targeting oxidative stress and inflammation [3]. Different classes of natural compounds, such as phenols, carotenoids, iridoids, and vitamins with antioxidant activity can be biosynthesized simultaneously by many plants. Phenols are well studied as natural antioxidants and are common components in the vegetable human diet [4]. At molecular level, carotenoids and flavonoids modulate inflammation as well as immunological processes.

Several epidemiological studies pointed to a significant correlation between a plant-based diet and a reduced risk of inflammation, while fat and animal products consumption increase the presence of inflammation markers in the plasma [5]. Furthermore, it has been reported that the prolonged activation of inflammatory cells generates Reactive Oxygen Species (ROS), inducing oxidative stress, which in turn, can damage DNA and tissues, making the organism more vulnerable to the emergence of several diseases, ranging from cardiovascular damage to diseases of central nervous system and alterations in the immune response [6]. Extraction process is a key step in the discovery of bioactive molecules from plants. Several extraction procedures commonly used include conventional methods namely maceration, percolation, decoction, infusion, etc. During the last decades, alternative techniques such as supercritical fluid extractions, microwave assisted solvent extraction, and ultrasound-assisted solvent extraction have gained increasing interest. These procedures showed some advantages as compared to the traditional methods. They are fast, environmentally friendly in terms of solvent and energy consumption [7,8]. However, extraction yields as well as biological effects of extracts obtained by using different extraction techniques have been reported to vary in different works [9,10].

*Arbutus unedo* L. (strawberry tree, Ericaceae family) is a plant of increasing interest because of its traditional, industrial, and medicinal use [11]. *A. unedo* has a circum-Mediterranean distribution, mainly in the coastal and inland areas with temperate climates, growing in north-eastern Africa, Canary Islands, western Asia, and Europe. In the Mediterranean basin, it is present in France, Italy, Albania, Greece, and Iberian Peninsula. *A. unedo* fruits (berries) are used for preparing alcoholic beverages, marmalades, jams, and jellies or are added to yoghurt. In traditional medicine, *A. unedo* fruits are known as antiseptic, diuretic and laxative agent, and to treat hypertension and kidney diseases. Roots, barks, and leaves are used as remedy for the treatment of hypertension, hypercholesterolemia, urological, dermatologic, gastrointestinal disorders, and vaginal infections [11]. Moreover, leaves are used for the treatment of diabetes and rheumatism. Flowers and stems are known as anti-inflammatory agents. The analysis of phytochemicals profile of fruits and leaves showed the presence of flavonoids, iridoids, anthocyanins, carotenoids, terpenoids, and fatty acids as the main classes of constituents [12,13]. The bioactivities of leaves and fruits of *A. unedo* have been reported in several studies [12,14,15,16,17]. Fruits have been investigated more than the leaves. Moreover, to the best of our knowledge, limited information about the comparison between the biological activities and the phytochemical contents of fruits and leaves extracts, obtained from fresh and dry matrix subject to various extraction methods, are provided from literature. To address to this issue, herein the in vitro biological activities as well as the phytochemical profile of twenty *A. unedo* extracts were investigated in order to define the compounds responsible of those activities. The choice of a suitable solvent in extraction of bioactive phytochemicals is considered as the most important step to recover these constituents [18]. No specific solvent is proposed for the optimum extraction of phytochemicals because of their various chemical properties that could modify their polarities and consequently influence their solvent solubility [19]. Therefore, the aims of this work were *i*) To obtain various extracts of leaves and fruits of *A. unedo* by different procedures adapted to the selective extraction of flavonoids and iridoids; *ii*) to identify these secondary metabolites by using a liquid chromatography-electrospray tandem mass spectrometry analysis (LC-ESI-MS/MS); and *iii*) to investigate the antioxidant, anti-inflammatory, and hypoglycaemic activities. 2,2-Diphenyl-1-picrylhydrazyl (DPPH), 2,2’-azino-bis-(3-ethylbenzothiazoline-6-sulfonic acid) diammonium salt (ABTS), ferric reducing antioxidant power (FRAP), and β-carotene bleaching tests were used to investigate the antioxidant properties. The hypoglycaemic activity of *A. unedo* extracts was studied evaluating their potential carbohydrates-hydrolysing enzymes (α-amylase and α-glucosidase) inhibitory activities. The anti-inflammatory potential activity was analysed by examining the concentrations of nitrite and nitrate that represent the final products of nitric oxide (NO) oxidation pathways.

## 2. Materials and Methods

### 2.1. Chemicals and Reagents

Solvents of analytical grade were purchased from VWR International s.r.l. (Milan, Italy). Solvents used for liquid chromatography-electrospray ionization-quadrupole-time of flight-mass spectrometry (LC-ESI-QTOF-MS) were purchased from Carlo Erba s.r.l. (Milan, Italy). Tween 20, ascorbic acid, Folin-Ciocalteu reagent, 3-(4,5-dimethyl-2-thiazolyl)-2,5-diphenyl-2H-tetrazolium bromide (MTT), Griess reagent, interleukin-2 (IL-2), sodium carbonate, butylated hydroxytoluene (BHT), propyl gallate, quercetin, ascorbic acid, 2,2-diphenyl-1-picrylhydrazyl (DPPH), 2,4,6-tripyridyl-s-triazine (TPTZ), 2,2′-azino-bis(3-ethylbenzothiazoline-6-sulfonic acid) diammonium salt, (ABTS) solution, *β*-carotene, acetic acid, linoleic acid, dimethyl sulfoxide (DMSO), Dulbecco’s modified Eagle’s medium, fetal bovine serum, glucose, penicillin-streptomycin, potato starch, sodium acetate, sodium phosphate, sodium potassium tartrate, sodium chloride, α-amylase from porcine pancreas (EC 3.2.1.1), α-glucosidase from *Saccharomyces cerevisiae* (EC 3.2.1.20), maltose, 3,5-dinitrosalicylic acid, *o*-dianisidine colour reagent (DIAN), and peroxidase-glucose oxidase (PGO) were purchased from Sigma-Aldrich s.r.l. (Milan, Italy). Gallic acid, protocatechuic acid, quinic acid, ferulic acid, ellagic acid, catechin, syringic acid, isoquercitrin, quercetin, rutin, and kaempferol were purchased from Sigma-Aldrich s.r.l. (Orleans, France). Geniposide, chlorogenic acid, and hyperoside were purchased from Extrasynthese (Lyon, France). Acarbose from *Actinoplanes* sp. was obtained from Serva (Heidelberg, Germany).

### 2.2. Plant Materials

Leaves and fruits of *Arbutus unedo* were collected from the Botanic Garden, University of Calabria, Rende (Cosenza, Southern Italy) (39°35′74″ N, 16°22′94″ E) (plant n. 103) in November 2016 and identified by Dr. N.G. Passalacqua, Natural History Museum of Calabria and Botanic Garden (CLU), University of Calabria (Rende, Italy). Samples were examined for integrity and absence of dust and insect contamination. Fruits were harvested at maturity stage, defined by visual colour (dark red) and size measurement.

### 2.3. Extraction Procedure

*A. unedo* leaves and fruits were investigated both as fresh and dried products. For this purpose, leaves (4.1 kg) and ripe fruits (5.9 kg) were separated in two parts. Leaves (1.8 kg) were dried at room temperature for 7 days in the dark. The drying of fruits (2.4 kg) was carried out in a gravity convection oven (Thermo Scientific Heraeus, Germany) at 50 °C for 7 days. During the drying process, the temperature was stable, and its distribution is based on warm air moving upwards. The benefit of this technology is very low air turbulences for gentle drying and heating. Daily determination of weight was measured with weight electronic scale until the weight was stable.

Plant materials (2.3 kg of fresh leaves and 674 g of dried leaves; 3.5 kg of fresh fruits and 950 g of dried fruits) were subjected to different exhaustive extraction procedure, namely a) maceration using ethanol (1 L, 3 × 72 h), and a solution 6:4 *v*/*v* EtOH/H_2_O (1 L, 3 × 72 h) as solvent; b) Soxhlet apparatus (conventional glass with an extraction chamber with a diameter of 8 cm and a height of 30 cm, accompanied by a flask of capacity of 1 L), using ethanol (600 mL, 7 cycles); c) ultrasound by using Branson 3800 ultrasonic system, series CPXH (130 W, 40 kHz frequency, Emerson, Milan, Italy), using ethanol as solvent (150 mL, 3 × 1 h); d) decoction (1:1 *w*/*v*, 30 min for fruits, 1:10 *w*/*v*, 10 min for fresh leaves, 1:20 *w*/*v*, 10 min for dried leaves). Extractive solutions, after being filtered and combined, were evaporated under reduced pressure in order to obtain dry extracts.

### 2.4. Total Iridoids Content (TIC)

The total content of iridoids (TIC) was determined according to a colorimetric method based on the Trim and Hill reaction. In this assay, 400 μL of extract (1.5 mg/mL) was mixed with 4.0 mL of Trim-Hill reagent (acetic acid/0.2% CuSO_4_/HClaq, 10:1:0.5; *v*/*v*/*v*). After the sample had been heated at 100 °C for 5 min, the absorption was read at 609 nm, a blue colour indicating the presence of iridoids. TIC was determined in triplicate and expressed as milligrams of aucubin equivalents (AU)/g of extract.

### 2.5. Total Phenols Content (TPC)

For the determination of the total phenols content (TPC), the Folin-Ciocalteu method was employed [20]. In brief, 100 μL of extract (1.5 mg/mL) was mixed with 2 mL of water, 1 mL of Na_2_CO_3_ 15% (*w*/*v*) aqueous solution, and 0.2 mL of Folin-Ciocalteu reagent. After 2 h of incubation at 25 °C, the absorbance was measured at 765 nm using a UV-vis Jenway 6003 spectrophotometer (Milan, Italy). TPC was determined in triplicate and expressed as milligrams of chlorogenic acid equivalents (CA)/g of extract.

### 2.6. Total Flavonoids Content (TFC)

The total flavonoids content (TFC) was determined as previously described [21]. In this assay, 1 mL of extract (1.5 mg/mL) was added to 4 mL of distilled water and 0.3 mL of 5% (*w*/*v*) sodium nitrite. After 5 min of reaction, 0.6 mL of 10% (*w*/*v*) AlCl_3_ was added, and 6 min later, 2 mL of 1 M NaOH and 2.1 mL of distilled water were added. Absorbance was read at 510 nm. The total flavonoids content was determined in triplicate and expressed as milligrams of quercetin equivalents (QE)/g of extract.

### 2.7. Liquid Chromatography-Electrospray Ionization-Quadrupole-Time of Flight-Mass Spectrometry (LC-ESI-QTOF-MS)

*A. unedo* extracts were solubilized in methanol, filtered, and analysed using an HPLC (U-3000, Thermo, Courtaboeuf, France) coupled to an ESI-QTOF mass spectrometer (Maxis II, Bruker, Champs sur Marne, France), as previously described [22] with some modifications as shown below. The chromatographic separation was performed on a C18 column (Acclaim RSLC polar advantage II, 100 × 2.1 mm, 2.2 μm) maintained at a temperature of 35 °C, with a speed of flow of 0.3 mL/min. The mobile phase consists of a mixture of 0.1% formic acid, 10% methanol and water (phase A), and 0.1% formic acid and acetonitrile (phase B). The elution gradient was as follows: 0 to 2 min 95% A; 2 to 7 min, 95 to 85% A; 7 to 15 min, from 85 to 50% A; 15 to 18 min, 50 to 20% A; 18 to 19 min, 20% and 19 to 21 min, 20 to 95% A. The injection volume was 2 μL and the flow rate was 0.3 mL/min. Chromatograms were acquired at four different wavelengths namely 240, 270, 340, and 510 nm. Mass spectra were acquired in positive mode by using the following parameters: ESI 3500 V, *m*/*z* 50–1200, MS 2 Hz. Compounds were identified based on UV spectra, and molecular weight (*m*/*z* ion [M+H]^+^ or [M+Na]^+^). The presence of quinic acid, ferulic acid, gallic acid, syringic acid, protocatechuic acid, chlorogenic acid, catechin, quercetin, isoquercitrin, ellagic acid, rutin, geniposide, hyperoside, and kaempferol was confirmed by using authentic standards.

### 2.8. Antioxidant Activity

#### 2.8.1. DPPH Radical Scavenging Activity Assay

The 2,2-diphenyl-1-picrylhydrazyl (DPPH) radicals scavenging activity was determined according to the method previously described [23]. Methanolic solutions of *A. unedo* extracts (200 μL, at concentration in the range 1–1000 μg/mL) and the DPPH methanol solution (800 μL, at concentration of 1.0 × 10^−4^ M) were prepared. The mixture was left in the dark at room temperature for 30 min. The absorbance was read at 517 nm. The positive control was ascorbic acid. The DPPH radicals scavenging activity was calculated as follows: [(A_0_ − A_1_)/A_0_] × 100, where A_0_ is the absorbance of the control and A_1_ is the absorbance in the presence of the samples.

#### 2.8.2. ABTS Radical Scavenging Activity Assay

In this assay, the 2,2′-azino-*bis*(3-ethylbenzothiazoline-6-sulfonic acid) diammonium salt (ABTS) solution was mixed with potassium persulfate and left in the dark for 12 h before use [23]. Samples (10 μL at concentration of 1–400 μg/mL in methanol) were added to the ABTS methanol solution (1 mL), and the absorbance was measured after 6 min. Ascorbic acid was the positive control. The ABTS radical scavenging ability was calculated with this equation: [(A_0_ − A_1_)/A_0_] × 100, where A_0_ is the absorbance of the control and A_1_ is the absorbance in the presence of the samples.

#### 2.8.3. β-Carotene Bleaching Test

This assay was done following the procedure previously described [24]. Concisely, *β*-carotene solution (1 mL) was added to 20 μL of linoleic acid and 200 μL of 100% Tween 20. After evaporation of chloroform and dilution with water (100 mL), the emulsion (5 mL) was mixed with 200 μL of extracts (1–100 μg/mL in methanol). Tubes were placed in a water bath at 45 °C. The absorbance was read at 470 nm against a blank at t = 0 and after 30 and 60 min of incubation. Propyl gallate was used as the positive control. The antioxidant activity (AA) was calculated as follow: AA = [(A_0_ − A_t_ )/(A_0_^*^-A_t_^*^) × 100], where A_0_ and A_0_^*^ are the absorbance values obtained at the time 0 for samples and control, respectively, while A_t_ and A_t_^*^are the absorbance values obtained after 30 and 60 min of incubation for samples and control, respectively.

#### 2.8.4. Ferric Reducing Activity Power (FRAP) Assay

This assay is based on the redox reaction that involves TPTZ-Fe^3+^ complex [23]. FRAP reagent was prepared by mixing 2.5 mL of 20 mM FeCl_3_, 25 mL of 0.3 M acetate buffer, and 2.5 mL of 10 mM TPTZ solution in 40 mM HCl. The methanol solution of each extract (200 μL at concentration of 2.5 mg/mL) was added to FRAP reagent (1.8 mL) and the absorption was measured at 595 nm. The positive control was butylated hydroxytoluene (BHT).

#### 2.8.5. Global Antioxidant Score (GAS) Calculation

For each *A. unedo* extract, the average of the T scores was used to calculate the value of the global antioxidant score (GAS). The T-score is calculated from the equation:

T-score = (X − min)/(max − min), where min and max represent the smallest and largest values, respectively, of the variable X between the studied extracts [25].

#### 2.8.6. Relative Antioxidant Capacity Index (RACI)

Relative antioxidant capacity index (RACI) is used as an integrated approach to evaluate and compare the antioxidant capacity of different samples [26]. Herein, data obtained from ABTS, DPPH, FRAP, and β-carotene bleaching tests were used to calculate RACI value for *A. unedo* samples. Standard scores were derived from data from different chemical methods without unrestricted units and no variance between the methods. The standard score is calculated using the following equation: RACI = (x − μ)/σ where x is the raw data, μ is the mean, and σ is the standard deviation.

### 2.9. Cell Viability Assay

#### 2.9.1. Cell Culture

Human Foreskin Fibroblast (HFF1), obtained from the American Type Culture Collection (Rockville, MD, USA), were cultured in Dulbecco’s modified Eagle’s medium (Sigma-Aldrich s.r.l., Milan, Italy) supplemented with 15% fetal bovine serum, 4.5 g/L glucose, 100 U/mL penicillin, and 100 μg/mL streptomycin. Cells were plated at a constant density to obtain identical experimental conditions in the different tests, and to achieve a high accuracy of the measurements.

#### 2.9.2. MTT Bioassay

The MTT assay was performed to assess the cells viability on a 96 multiwell plate (8 × 10^3^ cells/well). After 24 h of incubation in humidified atmosphere of 5% CO_2_ at 37 °C to allow cell attachment, cells were treated with different concentrations of *A. unedo* extracts (12.5–250 μg/mL).

This assay measures the conversion of tetrazolium salt to yield coloured formazan in the presence of metabolic activity. The amount of formazan is proportional to the number of living cells [27]. The optical density was measured with a microplate spectrophotometer reader (Titertek Multiskan, Flow Laboratories, Helsinki, Finland) at *λ* = 570 nm. Results are expressed as percentage cell viability with respect to control (untreated cells).

### 2.10. Measurement of Nitrite and Nitrate Concentration

Nitric oxide (NO) is as a potent mediator in several cellular processes such as regulation of neurotransmission, vascular tone, host defense mechanisms, and inflammation [28]. The use of NO inhibitors represent an important therapeutic approach in the management of inflammatory diseases. Herein, the inhibitory effects of *A. unedo* extracts on NO production were investigated by using the assay based on the reaction of diazocopulation of nitrite with the Griess reagent [29]. HFF1 cells, pre-treated with 12.5 μg/mL of sample for 90 min, were stimulated with interleukin-2β (IL-2β) (10 μg/mL) for 30 min. The method is based on the reaction of diazocopulation of nitrite with the Griess reagent. The total nitrite concentration in the cells was measured by adding 250 μL of Griess reagent to 250 μL of medium. The optical density of each well was measured with a microplate spectrophotometer reader (Titertek Multiskan, Flow Laboratories, Helsinki, Finland) at 546 nm. Results were calculated by comparison with OD_550_ of standard solutions of sodium nitrite prepared in H_2_O and expressed as percentage of nitrite production respect to untreated and interleukin stimulated cells.

### 2.11. In Vitro Evaluation of Hypoglycaemic Activity

#### 2.11.1. α-Amylase Inhibitory Activity Assay

The α-amylase inhibition assay was performed as previously described [30]. Briefly, 25.3 mg of enzyme in 100 mL of cold distilled water was prepared. The starch solution was prepared with 125 mg of potato starch in 25 mL of sodium phosphate buffer 20 mM and sodium chloride 6.7 mM, at 65 °C for 15 min. The colorimetric reagent was prepared by mixing a sodium potassium tartrate solution (24 g of sodium potassium tartrate in 16 mL of sodium hydroxide 2 M) and 96 mM 3,5-dinitrosalicylic acid solution (0.88 g of acid in 46 mL of water). The extracts (40 μL at concentration in the range 12.50–1000 μg/mL) and control were added to the starch solution and left to react with enzyme at 25 °C. The absorbance was read at 540 nm. Acarbose was used as the positive control.

#### 2.11.2. α-Glucosidase Inhibitory Activity Assay

In α-glucosidase inhibition assay a maltose solution was prepared mixing 12 g of maltose in 300 mL of 50 mM sodium acetate buffer [30]. Total of 1 mg of enzyme in 10 mL of ice-cold distilled water was prepared for the enzyme solution. *o*-Dianisidine colour reagent (DIAN) solution was prepared by dissolving 1 tablet in 25 mL of distilled water. Peroxidase-glucose oxidase (PGO) system-colour reagent solution was obtained by dissolving 1 capsule in 100 mL of ice cold distilled water. Samples (5 μL at concentration in the range 12.50–1000 μg/mL) and control were stirred to maltose solution and left to equilibrate at 37 °C. The reaction was started with addition of α-glucosidase solution. The reaction was stopped by adding a solution of perchloric acid after 30 min of incubation at 37 °C. The supernatant of tube of step one was mixed with DIAN and PGO and was left to incubate at 37 °C for 30 min. The absorbance was read at 540 nm. Acarbose was used as the positive control.

### 2.12. Statistical Analysis

The concentration giving 50% inhibition (IC_50_) was calculated by nonlinear regression with the use of Prism GraphPad Prism, version 4.0 for Windows (GraphPad Software, San Diego, CA, USA). The concentration-response curve was obtained by plotting the percentage inhibition vs. concentration. One-way analysis of variance test (ANOVA) followed by a multi-comparison Dunnett’s test were applied. Pearson’s correlation coefficient (*r*) and linear regression, assessment of repeatability, calculation of average and relative standard deviation was performed using Excel 2010 software (Microsoft, Washington, USA).

## 3. Results and Discussion

The chemical composition of plants, quantity, and availability of bioactive compounds depends on various factors, including climatic conditions, soil, and seasonal harvest. Among the various extractive methods to recover phytochemicals from natural materials, some of them preserve thermolabile compounds such as maceration, while others at higher temperatures lead to better extraction efficiency, but sometimes lead to degradation of heat-unstable compounds. Thus, the various techniques associated with the nature of the extractive solvents used are capable of modifying not only the extraction yields but also the composition of the extracts and consequently their biological effects. The extracts used in therapeutics are aqueous, ethanolic, or alcoholic (EtOH/H_2_O). These solvents are known to solubilize both phenols and iridoids.

### 3.1. Effects of Extractive Methods on Extraction Yield and Phytochemical Contents

**Figure 1:**
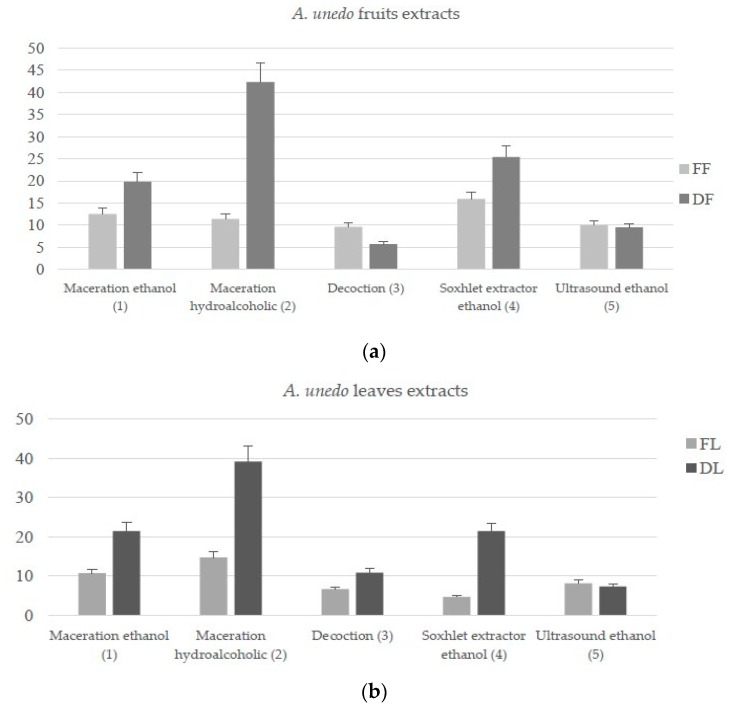
Extraction yield (%) of *A. unedo* fruits (**a**) and leaves (**b**) extracts. FF: fresh fruits; DF: dried fruits; FL: fresh leaves; DL: dried leaves.

Herein, four methods for the extraction of bioactive components from the leaves and fruits of *A. unedo*, were used (Figure 1). The best extraction yield was obtained with hydroalcoholic maceration of dried materials with values of 42.3 and 39.2% for fruits (DF2) and leaves (DL2), respectively. With fresh matrix, the best technique is the extraction of fruits by using Soxhlet apparatus (15.9%; FF4) and the hydroalcoholic maceration of leaves (14.7%; FL2).

Less extraction yield was observed with other extractive methods. Contrary to the performance observed with other techniques used, ultrasound give similar yields with the fresh than dry matrix, both for the leaves (8.1 and 7.3% respectively for fresh (FL5) and dried (DL5) extracts) and fruits (10.1 and 9.5% respectively for fresh (FF5) and dried (DF5) extracts). Only the decoction of fresh fruits furnished a more yield than the dried fruits (9.6 and 5.7% respectively for fresh (FF3) and dried (DF3) extracts). In a previous work, Oliveira et al. [15] reported water as more efficient in the extraction of *A. unedo* leaves (32.1%) compared with ethanol (15%). On the other hand, Orak et al. [16] showed ethanol (39.6%) as a better extraction solvent in comparison with water (38.93%). Isbilir et al. [31] confirmed that ethanol (70.3%) has greater efficacy as a solvent than water (50.3%). Moreover, in an efficient way, different matrices, such as fresh and dried leaves and fruits, allowed the extraction of different classes of phytochemicals. The total content of some classes of compounds, namely polyphenols, flavonoids, and iridoids, has been evaluated (Table 1).

The content of each group of chemical metabolites is quite similar in both fresh and dried extracts. Leaves have a higher total phenols content (173~376 mg CA equivalents/g extract), compared to the fruits (34~82 mg CA equivalents/g extract). The same trend was observed for flavonoids (83~190 mg and 25~29 mg QE equivalents/g extract for leaves and fruits, respectively). Analysing the results obtained with fresh leaves, interesting results were obtained by decoction (FL3) and ethanol maceration (FL1), with values of 376.01 mg CA equivalents/g of extract and 178.67 mg QE equivalents/g of extract for total phenols and flavonoids content, respectively. There was no substantial difference in the iridoids content between leaves and fruits. The richest extract in iridoids was obtained by ultrasound (220.14 mg AU equivalents/g of extract, FL5).

Among the extracts from dried leaves, maceration with hydroalcoholic solution (DL2) presented the best content of polyphenols and iridoids (329.33 and 170.67 mg/g, respectively). The ethanolic extracts obtained by ultrasound (DL5) and Soxhlet apparatus (DL4) were rich in flavonoids (190.04 mg QE equivalents/g extract).

The hydroalcoholic maceration of fresh fruits (FF2) allowed obtaining the highest content in polyphenols and flavonoids (respectively values of 40.06 and 29.13 of extract). The higher content in iridoids was obtained with ethanol extraction using the Soxhlet apparatus (FF4) (158.67 mg AU equivalents/g extract). Among the dried fruit samples, the total polyphenols content was present in ethanol maceration (DF1) and decoction (DF3) with values of 82.20 and 81.73 mg/g of extract respectively. Interesting is the data related to the total content in iridoids reporting the following trend DF5> DF2 > DF4> DF1> DF3. Our values are in accordance with the results described by Bouzid et al. [32], content in polyphenols and flavonoids from aqueous fruits extract measure out to lower values (12.75 and 2.18 mg/g respectively). Moreover, results obtained by Salem et al. [33] highlight the hydroalcoholic maceration of the fruits as the best technique to obtain the highest content of bioactive compounds.

### 3.2. Phytochemicals Identification

Previous studies on *A. unedo* showed that phenols and iridoids are well extracted by using alcoholic solutions [34,35], anthocyanins by using methanol solution of HCl [36], and apolar compounds such as saturated fatty acids and carotenoids by employing acetone-petroleum ether mixture. Carotenoids were not soluble in the methanol and for the preservation of vitamin C and *β*-carotene, the extract needs to be freeze-dried rapidly because the sensitivity to oxidation is known. The nyacin was only extracted by H_2_SO_4_ (0.5 M) [37]. LC-ESI-QTOF-MS analyses showed the presence of phenolic acids, iridoids, proanthocyanidins, and flavonoids as main constituents of both leaves and fruits (Table 2 and Table 3). Among these compounds, quinic acid, ferulic acid, gallic acid, syringic acid, chlorogenic acid, protocatechuic acid, catechin, isoquercitrin, ellagic acid, rutin, geniposide, hyperoside, and kaempferol were confirmed with authentic standards. Other compounds were identified based on UV spectra, and molecular weight (*m*/*z* ion [M+H]^+^ or [M+Na]^+^). The number of phenolic compounds identified in the leaves was higher than in fruits extracts, as confirmed in the literature [38]. The structures of the compounds were determined on the basis of the spectral data previously described [39,40,41].

#### 3.2.1. Phenolic Acids and Phenolic Glucosides

**Figure 2:**
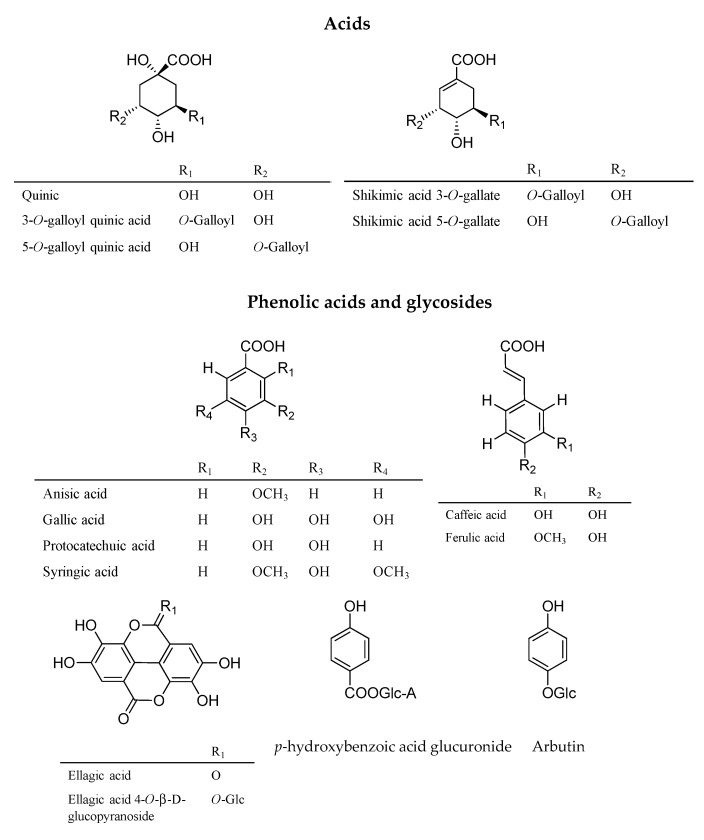
The main acids, phenolic acids, and phenolic glycosides identified in *A. unedo* extracts.

Fruits and leaves extracts are characterized by the presence of various phenolic acids (Figure 2). Quinic acid, anisic acid, ellagic acid 4-*O*-β-D-glucopyranoside, shikimic acid gallate, gallic acid, ferulic acid, and galloyl quinic acid were systematically present. Caffeic acid, ellagic acid, and *p*-hydroxybenzoic acid glucuronide were identified only in the leaves extracts. Moreover, arbutin and caffeic acid were specifically detected in dried leaves extracts obtained by maceration with ethanol and ethanol/water (6/4), according to the literature [13]. The majority of phenolic acids identified were previously described [36,42,43,44,45,46].

Syringic acid was detected in all leaves extracts and only in the macerations of fresh fruits and Soxhlet extract of dried fruits. The protocatechuic acid was found only in fruits extracts (FF1, FF2, FF3, and DF1). Quinic, ferulic, caffeic acids, and ellagic acid 4-*O*-β-D-glucopyranoside were first found in *A. unedo*.

#### 3.2.2. Flavonoids

**Figure 3:**
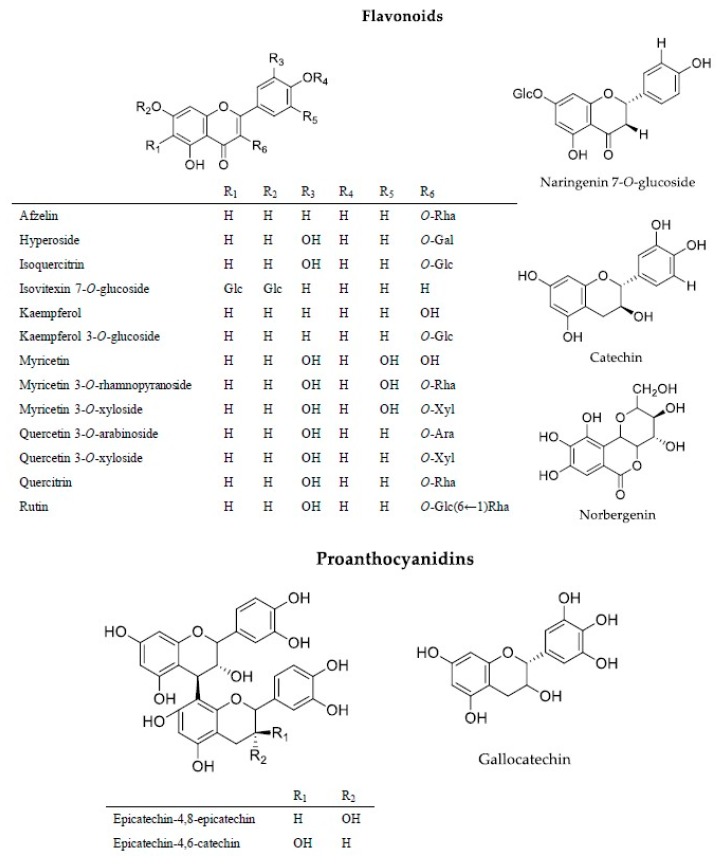
Principal flavonoids identified in *A. unedo* extracts.

Flavonoids are identified in all *A. unedo* extracts. However, there are difference in dependence of the extraction processes. Quercetin derivatives (arabinoside, xyloside, and rhamnoside) were detected in all extracts (Figure 3). Afzelin, kaempferol 3-*O*-glucoside, naringenin 7-*O*-glucoside, rutin, isovitexin 7-*O*-glucoside, myricetin 3-*O*-xyloside, and kaempferol were found only in the leaves extracts. Norbergenin was found only in the dried leaves extracts obtained by maceration with ethanol and ethanol/water. Isoquercitrin, hyperoside, and myricetin 3-*O*-rhamnopyranoside characterized all leaves extracts and dried fruits extracts; instead catechin was found in all leaves extracts and ethanol extract obtained after maceration of fresh fruits. 

Myricetin was observed in all extracts obtained by fruits and only in the extract obtained by dried leaves maceration, decoction, and Soxhlet extracts of fresh leaves. To the best of our knowledge, naringenin 7-*O*-glucoside, kaempferol 3-*O*-glucoside, isovitexin 7-*O*-glucoside, myricetin 3-*O*-rhamnopyranoside, norbergenin, and myricetin were identified for the first time in *A. unedo* extracts.

#### 3.2.3. Proanthocyanidins

In contrast to the literature, proanthocyanidins detected in the present study were identified in the leaves, but not in the fruits [47]. In particular, epicatechin-4,6-catechin and epicatechin-4,8-epicatechin were found in all leaves extracts, while gallocatechin was identified in some extracts (FL3, FL4, DL1-3) (Figure 3).

#### 3.2.4. Iridoids

**Figure 4:**
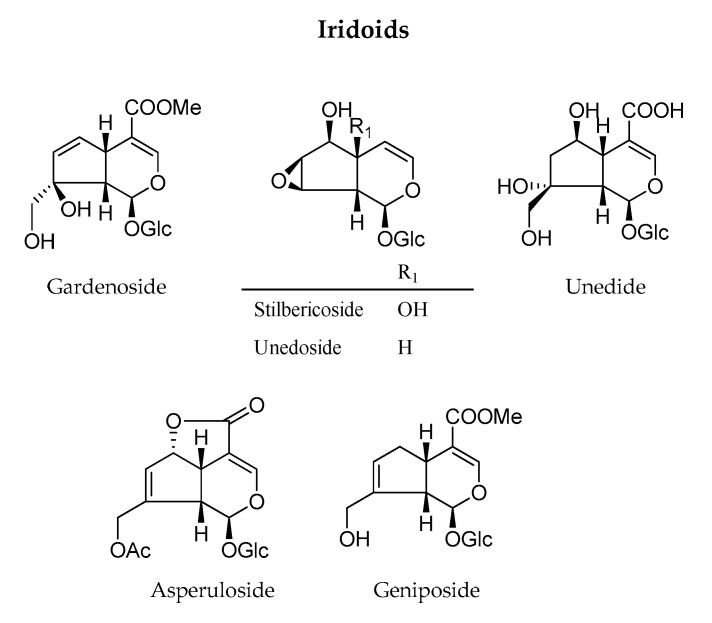
Iridoids identified in *A. unedo* extracts.

Six iridoids were identified in *A. unedo* leaves and fruits extracts (Figure 4). Three of these compounds, namely gardenoside, geniposide, and unedoside, are specifically produced by the leaves, while all have been found in fruit extracts. Analysing results obtained with fruits extracts, some interesting differences can be highlighted. Stilbericoside (except for FF1) was identified in extracts from dried fruits while unedide in fresh fruits. Interestingly, asperuloside was extracted only from dried fruits by ethanol with Soxhlet apparatus (DF4).

Geniposide and unedoside were not extracted from fresh leaves but only by ethanol (DL1) and hydroalcoholic maceration (DL2) of dried leaves. Stilbericoside was detected only in the decoction of dried materials (DL3). Unedide was detected in all dried leaves extracts and in a decoction of fresh leaves (FL3). Asperuloside and gardenoside were found in fresh and dried extracts. Asperuloside were not only extracted by ethanolic (DL1) and hydroalcoholic (DL2) macerations but also by decoction of the fresh leaves (FL2). Few works in the literature have investigated the presence of *A. unedo* iridoids. The isolation of asperuloside, geniposide, gardenoside stilbericoside, unedoside, and unedide was reported [34,48]. One of these studies is that carried out by Davini et al. [48], which isolated unedide and monotropein from the ethanol (90%) extract of the leaves of *A. unedo*. Unedoside is an iridoid rarely biosynthesized in plant kingdom and it is considered as a chemotaxonomic marker of *Arbutus* genus [13].

### 3.3. Antioxidant Activity

The increasing interest gained by antioxidants is due to the health benefits provided mainly by natural compounds preventing the occurrence of oxidative-stress related diseases, caused by the attack of free radicals on key bio-components like lipids or nucleic acids. Several methods were recently developed for measuring the antioxidant capacity of a sample. These tests vary in the mechanism of generation of different target molecules and/or radicals and in the way end-products are measured. Considering that different antioxidants may act in vivo through different mechanisms of action to investigate the antioxidant activity choosing adequate assays is critical and no single method can fully evaluate the antioxidant activity of a sample [49].

Therefore, in the present study, four assays namely ABTS, DPPH, FRAP, and β-carotene bleaching tests were used to investigate and compare the antioxidant potential of *A. unedo* extracts.

Results are reported in Table 4.

*A. unedo* extracts were tested for their potential free radicals scavenging activity by using ABTS and DPPH assays. ABTS test measures the ability of an antioxidant to scavenge the ABTS radicals that are produced in aqueous phase by the reaction of the ABTS salt with a strong oxidizing agent (potassium persulfate or potassium permanganate). The reduction of blue-green ABTS coloured solution by hydrogen-donating antioxidant is measured by the suppression of its characteristic long wave absorption spectrum. DPPH is a stable purple free radical because of the delocalization of the spare electron on the whole molecule. When DPPH radical reacts with a hydrogen donor, the reduced form is generated, accompanied by the disappearance of the violet colour.

All extracts showed antioxidant properties in a concentration-dependent manner. In ABTS test, the best results were obtained with dried leaves. The most active extracts were obtained by maceration with ethanol (DL1) and the hydroalcoholic solution (DL2) with IC_50_ values of 0.39 and 0.42 μg/mL, respectively. The same trend was observed in DPPH test with IC_50_ values of 3.94 and 0.98 μg/mL, respectively. Instead, different results have been obtained for the extracts of fresh leaves. The extract obtained by decoction (FL3) displayed high antioxidant activity against ABTS radicals with an IC_50_ value of 1.16 μg/mL, while extracts obtained by both ethanol (FL1) and ultrasound (FL5) maceration were the most active toward DPPH radicals with IC_50_ values of 6.89 μg/mL.

*A. unedo* dried fruits were more active than fresh fruits. The best anti-radicals activity was found by extract obtained with Soxhlet (DF4) apparatus (IC_50_ value of 1.16 μg/mL) in ABTS test and by decoction (DF3; IC_50_ value of 32.21 μg/mL) in DPPH test. The same trend was observed in DPPH test with IC_50_ values of 3.94 and 0.98 μg/mL, respectively for DL1 and DL2. Instead, different results have been obtained for the extracts of fresh leaves. The extract obtained by decoction (FL3) displayed the high antioxidant activity against ABTS radicals with an IC_50_ value of 1.16 μg/mL, while extracts obtained by both ethanol (FL1) and ultrasound (FL5) maceration were the most active toward DPPH radicals with IC_50_ values of 6.89 μg/mL.

Several studies that investigated the antioxidant properties of *A. unedo* leaves are present in literature. Some of these assessed the influence of the extraction solvent on the antioxidant activity [15,16,38]. Oliveira et al. [15] used different solvent (ethanol, methanol, water, and diethyl ether) for the extraction of leaves and evaluated the in vitro antioxidant activity. In the DPPH test, the extract more active was ethanol extract with IC_50_ of 63.2 μg/mL, followed by water extract with IC_50_ of 73.7 μg/mL. In the superoxide anion test, with IC_50_ of 6.9 μg/mL, methanol extract showed high potential. Whereas, the extract in ethyl ether had no antioxidant activity in any antioxidant tests. For Orak et al. [16] the aqueous extract had the highest content in total polyphenols (197.16 mg/g) compared to the methanol and ethanol extracts (169.05 mg/g and 119.97 mg/g, respectively). Contrary to the expectations, the ethanol extracts are more active in inhibiting the DPPH radical with an IC_50_ of 0.423 mg/mL. Previously, Mendes et al. [38] studied the antioxidant activity of leaves aqueous extract with different in vitro assays. Data obtained showed that the antioxidant activity was correlated with high phenolic content (170 mg/g), as demonstrated by DPPH test (0.087 mg/mL). This result was further confirmed by the effect on hemolysis of erythrocyte (IC_50_ of 0.062 mg/mL) [38]. Orak et al. [17] reported the antioxidant activity of *A. unedo* fruits fresh and after drying treatment. Generally, fresh fruits exhibited high antioxidant activity compared to dried fruits. Mendes et al. [38] reported also the phenolic content (16.7 mg/g) and antioxidant properties of aqueous extract of *A. unedo* fruits. The extract presented an IC_50_ value of 0.79 mg/mL in the DPPH test. In addition, the antihemolytic activity and lipid peroxidation inhibitory activity reported a value of IC_50_ of 0.43 and 0.73 mg/mL, respectively.

A recent study demonstrated the potential antioxidant activity for ethanol extract of fruits with value of IC_50_ of 324.06 and 515.76 μg/mL for respectively DPPH and ABTS tests correlated with high content of polyphenol (204.5 mg/g) and flavonoid (34.18 mg/g) total. While, the methanol extract showed the lower value of polyphenol and flavonoid (180.75 and 28.81 mg/g, respectively), with IC_50_ value of 379.50 and 523.87 μg/mL for DPPH and ABTS test [33].

β-Carotene bleaching test was used to investigate the ability of *A. unedo* extract to inhibit the lipid peroxidation. Maceration with ethanol of dried leaves (DL1) lead to the most active extract as inhibitor of lipid peroxidation with IC_50_ values of 1.85 and 4.09 μg/mL, respectively, after 30 and 60 min of incubation. For the fresh leaves after 30 min of incubation, the hydroalcoholic maceration (FL2) was indicated for the best IC_50_ value of 13.19 μg/mL; while after 60 min the ultrasound (FL5) extract demonstrated a better inhibition of lipidic peroxidation with IC_50_ of 7.94 μg/mL. Interesting data were obtained with the maceration in ethanol (DF1) of dried fruits with IC_50_ values of 2.54 and 4.81 μg/mL, respectively after 30 and 60 min of incubation. Orak et al. [16] have tested preventing the lipid peroxidation of leaves extracts with distinct solvents (water, methanol, and ethanol). The ethanol extract, with an IC_50_ of 0.666 mg/mL, is more active compared to methanol and aqueous extracts.

**Figure 5:**
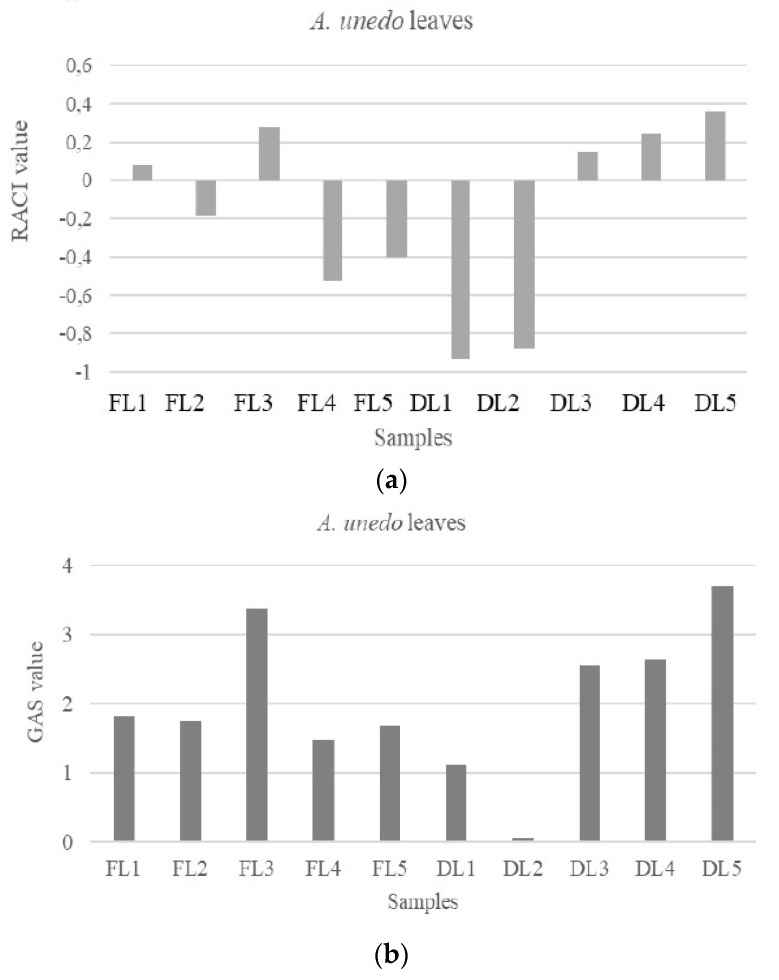
Evaluation of total antioxidant activity of leaves through Relative Antioxidant Capacity Index (RACI) (**a**) and Global Antioxidant Score (GAS) (**b**). FL: fresh leaves; DL: dried leaves. 1. Ethanolic maceration; 2. hydroalcoholic maceration; 3. decoction; 4. Soxhlet (EtOH) extraction; 5. ultrasound (EtOH) extraction.

Previously described by Mendes et al. [38], the aqueous extracts of leaves and fruits prevent lipid peroxidation with IC_50_ of 0.075 and 0.732 mg/mL, respectively. Our results are in agreement with these data regarding dried fruits decoction. On the contrary, much better values have been obtained in our study in relation to the leaves. The antioxidant properties of *A. unedo* samples were also assessed by using FRAP test. The used methods have different reaction mechanisms. For instance, ABTS and DPPH tests are based on electron and H atom transfer, while the FRAP test is based on electron transfer reaction. Except for FL3, extracts obtained from fresh leaves exhibited the highest activity in the FRAP test with values in the range 83.03–94.20 μM Fe (II)/g. These results highlight the greater capacity of fresh leaves to reduce iron. Oliveira et al. [15] used different solvents (ethanol, methanol, water, and diethyl ether) for the extraction of leaves and evaluated in vitro antioxidant activity. The more active extracts were obtained from ethanol, with IC_50_ of 232.7 μg/mL, followed by water with IC_50_ of 287.7 μg/mL. Previously, Mendes et al. [38] found the antioxidant activity of leaves and fruits aqueous extracts with IC_50_ value of 0.318 mg/mL and 2.89 mg/mL, respectively. GAS and RACI approaches were used to select the extract with the best antioxidant activity (Figure 5). Among fruits extracts, FF3 (GAS = 1.40; RACI = −0.44) and DF4 (GAS = 1.19; RACI = −0.58) samples showed the highest antioxidant potential.

Among the leaves extracts the highest activity was attributed to DL2 (GAS and RACI values of 0.07 and −0.88, respectively), and DL1 (GAS = 1.11; RACI = −0.93). The most active antioxidant extracts were obtained by ethanol and hydroalcoholic maceration of dried leaves. Interestingly, only in these extracts arbutin, caffeic acid, unedoside, geniposide, norbergenin were identified. Different studies aimed at investigating the antioxidant potential of these compounds are present in the literature. In the DPPH radicals scavenging assay, norbergenin exhibited an IC_50_ value of 13 μM [50], 11.2 μg/mL [51], and an inhibition of 85% [52], respectively. Caffeic acid is one of the hydroxycinnamate and phenylpropanoid metabolites are more commonly distributed in medicinal plants and foods. It is known to possess antioxidant activity in vitro [53,54,55]. Recently, in the DPPH test, Sidoryk et al. [56] showed an IC_50_ value of 32.2 μM. This compound was tested also in ABTS and FRAP tests, with percentage of 29.8 and 26.8%, respectively [55]. While Gülçin [54] reported a value of 92.9 and 53.2%, respectively for ABTS and FRAP tests. Arbutin is the monoglucoside of hydroquinone that is known as the potent antioxidant compound with two oxidizable hydroxyl groups in its structure. Arbutin retains one of these hydroxyl groups. Some studies showed that arbutin has antioxidant activity but not strong as its aglycone [57]. Takebayashi et al. [58] demonstrated that arbutin possessed weak but long-lasting radicals-scavenging effects and strong antioxidant activity comparable or superior to that of its aglycone in two cell-based antioxidant tests using skin fibroblasts and erythrocytes. As reported in several studies, generally the antioxidant activity of phenolic compounds was linked to hydroxyl groups present in their structure [55]. The phenylpropanoids act as antioxidant agents by chelating pro-oxidant metal ions especially iron and by eliminating free radicals [59]. The hydroxyl groups of these compounds confer antioxidant activity. However, there are other factors in determining the potency of their effects. The presence of a second hydroxyl group in the ortho position is known to increase the antioxidant activity due to an additional resonance stabilization and formation of o-quinone. This characteristic can be used to explain the antioxidant efficiency of caffeic acid. In the study of Gálvez et al. [60] the antioxidant activity of rutin, verbascoside, aucubin, and geniposide were evaluated using DPPH test. Rutin and verbascoside showed the highest antioxidant activity with IC_50_ values of 9.5 and 11.52 μM, respectively. Instead, aucubin and geniposide do not present DPPH radicals scavenging activity.

### 3.4. Inhibitory Effects on Nitric Oxide (NO) Production

**Figure 6:**
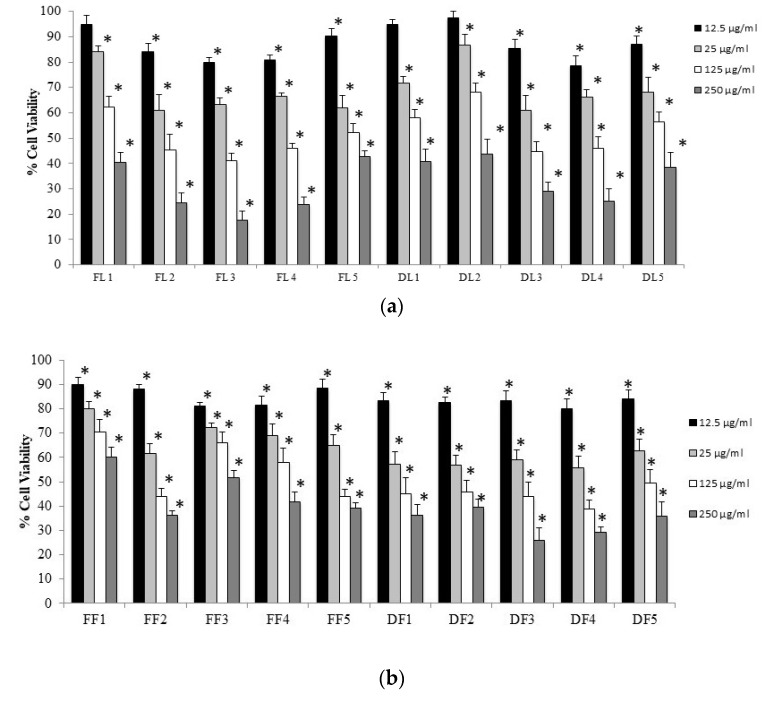
Cell viability in HFF1 cells untreated and treated for 24 h with leaves (**a**) and fruits (**b**) extracts of *A. unedo* at different concentrations (12.5–250 μg/mL). Values are the mean ± S.D. of four experiments in triplicate. * Significant vs. untreated control cells and vs. other concentrations of the same extract *p* < 0.001, *p* < 0.001.

Nitric oxide (NO) is recognized as a potent signaling mediator in several cellular processes. It is crucial in the regulation of neurotransmission, vascular tone, host defence mechanisms, and inflammation [28]. Therefore, NO inhibitors may represent important therapeutic agents in the management of inflammatory diseases. In this study, the beneficial effects of *A. unedo* extracts on the inhibition of the production of NO was evaluated in fibroblasts (HFF1 cells). Both *A. unedo* leaves and fruits extracts showed a cytotoxic effect in a concentration-dependent manner, decreasing cell viability of HFF1 cells independently of the extraction technique used (Figure 6).

**Figure 7:**
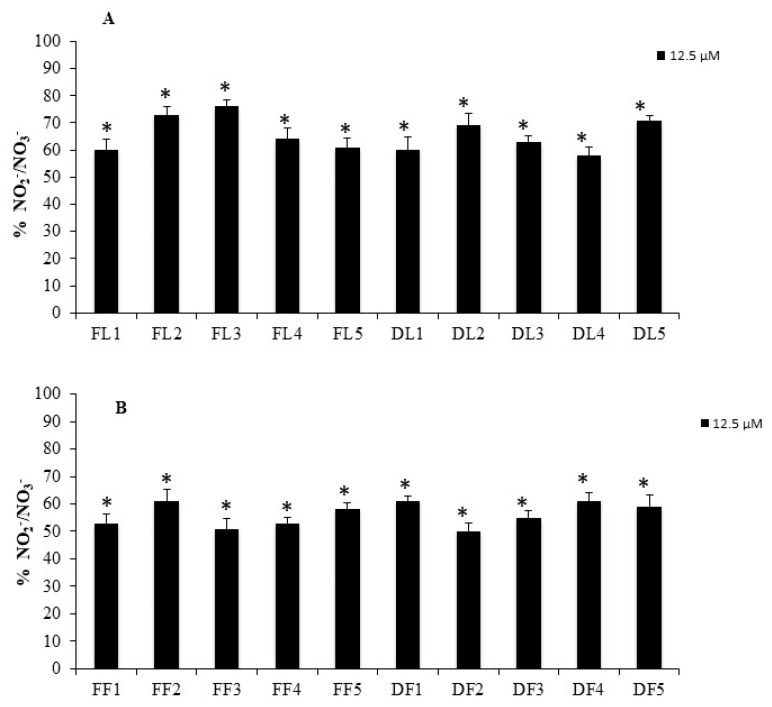
NO_2_^−^/NO_3_^−^ levels in HFF1 cells treated with leaves (**A**) and/or fruits (**B**) extracts of *A. unedo* at 12.5 μg/mL and stimulated with IL-2β (10 μg/mL). Values are the mean ± S.D. of four experiments in triplicate. *Significant vs. IL-2β treated control cells: *p* < 0.001.

Fruit extracts showed slight toxicity with respect to the leaves extracts. Inside the different groups of leaves/fruits and fresh and dried, alcoholic and hydroalcoholic maceration exhibited the highest ability in decreasing mitochondrial dehydrogenases activity. The lower concentration of 12.5 μg/mL of each extract was used as pre-treatment to perform the anti-inflammatory activity of each extract. As shown in Figure 7, pre-treatment with all different extracts were able to significantly reduce the nitrite formation.

No significant differences were observed inside the group of leaves or fruits extracts but all the fruits extract showed a more anti-inflammatory activity with respect to the leaves extract, in accordance with the previously reported by other authors [11,61]. It is known that increased NO formation contributes to the inflammatory process. Therefore, in our study, we demonstrated the reduction of levels of nitrites and nitrates (final metabolites of NO in water) induced by the treatments with *A. unedo* extracts, suggesting that the compounds present anti-inflammatory properties probably related to the phytocomplex. In addition, the reduction in the levels of nitrites and nitrates is also due to the inhibition of *i*NOS expression/activity or of some targets involved in inflammation, as reported for other species [62].

### 3.5. Hypoglycaemic Activity

Oxidative stress is one of the main inducers of *β*-pancreatic cells damage at the basis of the pathogenesis of diabetes. β-pancreatic cells are more sensitive, than other types of cells, to oxidative stress [63]. Cell decline play an important role in the development of diabetes mellitus [64].

Diabetes are characterized by chronic hyperglycaemia caused by the absolute or relative lack of insulin secretion or caused by insufficient insulin activity, which involve many clinical complications such as hyperlipidaemia, hyperinsulinemia, hypertension, and atherosclerosis, but also death if not controlled. The study of plant compounds seems to open a new line of research for the treatment of this pathology. One of the strategies used to reduce postprandial hyperglycaemia is the reduction or inhibition of carbohydrate absorption by inhibiting digestive enzymes such as α-amylase and α-glucosidase. Data are summarized in Table 5.

Among the extracts of fresh leaves, those obtained by ultrasound (FL5; IC_50_ of 19.56 μg/mL) and hydroalcoholic maceration (FL2; IC_50_ of 31.38 μg/mL) were the most promising as inhibitors of the α-glucosidase. These values are better than those relating to the positive control acarbose.

The extract obtained by ethanol maceration of fresh leaves (FL1) exhibited the highest activity in inhibiting α-amylase (IC_50_ value of 63.43 μg/mL).

Interesting results were obtained also by fresh fruits hydroalcoholic extract (FF2) with IC_50_ value of 28.42 μg/mL against α-glucosidase followed by ultrasound maceration (FF5) of fresh fruit (IC_50_ value of 40.25 μg/mL). Among the extracts from dried fruits sample DF4 showed the highest activity against α-amylase with an IC_50_ value of 77.51 μg/mL.

To the best of our knowledge, no previous works analysed strawberry tree leaves and fruits as carbohydrates-hydrolysing enzymes inhibitory agents. Some studies reported the potential activity of *A. unedo* roots as antidiabetic agents. Bnouham et al. [65] showed in vivo a decrease of the levels of glucose after co-administration of glucose and water extract of *A. unedo* roots in the oral glucose tolerance test (OGTT) but no in intravenous glucose tolerance test (IVGTT). The same trend was observed with oral administration of glibenclamide. More recently, Mrabti et al. [66] studied the hypoglycaemic properties of *A. unedo* roots finding an interesting inhibitory capacity of α-glucosidase (IC_50_ of 94.81 μg/mL) more efficient than the positive control (IC_50_ of 199.53 μg/mL).

### 3.6. Correlation between Phytochemicals Content and Bioactivity

*Pearson’s* correlation coefficient was used to describe the correlation between the biological activities and the content of components (TPC, TFC, and TIC). The following matrices were considered: fresh fruits, dried fruits, fresh leaves and dried leaves. Generally, as inferable from the data on the correlation analysis, the antioxidant potential measured by ABTS, DPPH, FRAP, and β-carotene bleaching assays, essentially follow the differences in content of total phenols, that is, the increase of the concentration of phenols corresponds to an increase of the antioxidant activity of the fruits extracts. However, a good correlation with the antioxidant activity of extracts was also appreciable with respect to TIC. In fact, a strong positive correlation was found between TIC and *β*-carotene bleaching test after 30 and 60 min of incubation for fresh fruits (*r* = 0.80 and 0.94, respectively). An *r* value of 0.73 was found for the TIC and DPPH test. In addition, fresh fruits TFC strongly positively correlated with α-amylase inhibitory activity (*r* = 0.93). Moreover, in fresh fruits a positive correlation was observed also for α-glucosidase inhibitory activity and TIC (*r* = 0.87).

Considering the fresh leaves extracts, the statistical analysis reveals a good correlation between the antioxidant activity (β-carotene bleaching test at 30 min of incubation) and TFC (*r* = 0.88), less relevant for TPC (*r* = 0.50), whereas no correlation was evidenced for TIC (*r* = −0.03). The same trend was observed with dried leaves. Of interest is also the correlation between the α-amylase inhibitory activity of both fresh and dried leaves extracts and TIC (*r* = 0.82 and 0.84, respectively). Overall obtained results showed that the significant difference of antioxidant capacity of *A. unedo* extracts was not related only to TPC, but also to other constituents such as flavonoids and iridoids.

## 4. Conclusions

Recently, the interest on natural antioxidants is growing. Plant-foods and medicinal plants represent a rich source of these active molecules. Among antioxidant compounds, especially flavonoids and iridoids exhibited a potential to protect cells from oxidative damage. Considering their interesting healthy properties, the choice of efficient extraction methods is drawing great attention. In this context, this study revealed a notable impact of different solvents and extraction procedures on yield extraction and phytochemicals composition, as well as the bioactivity of *A. unedo* leaves and fruits both fresh and dried. The novelty of this work is to have focused attention on two classes of phytochemicals such as flavonoids and iridoids previously poorly investigated in this species unlike anthocyanins. Considering all results, maceration gives extracts that preserve bioactive compounds showing high biological activity compared with other extractive techniques.

The extracts obtained by ethanolic and hydroalcoholic macerations of dried leaves presented the highest antioxidant activity with GAS values of 1.11 and 0.07, respectively, and were characterized by the presence of some secondary metabolites (arbutin, caffeic acid, unedoside, geniposide, and norbergenin) only found by using those extraction procedures.

The leaves extracts showed high antioxidant and hypoglycaemic activities compared with fruits extracts. Surprisingly, in the radicals scavenging and FRAP tests, leaves extracts were more active compared with the positive control.

Fruit extracts present high anti-inflammatory activity and a moderate toxicity compared with the leaves extracts. Moreover, alcoholic and hydroalcoholic maceration of extracts exhibited the highest ability in decreasing mitochondrial dehydrogenases activity. The LC-ESI-Q-TOF-MS allows finding compounds that are chemotaxonomic markers of the *Arbutus* genus and for the first time ellagic acid 4-*O*-β-D-glucopyranoside, kaempferol 3-*O*-glucoside, and norbergenin.

Our current findings demonstrated that leaves and fruits extracts of *A. unedo* could be used as a source of bioactive iridoids and phenolics compounds, that contribute to different biological activities attributed to extracts. Future studies should focus on the contribution of the identified compounds to the biological activity to prospect a potential of *A. unedo* extracts as nutraceuticals and/or functional foods.

## Figures and Tables

**Table 1:** Total phenols, flavonoids, and iridoids content of *A. unedo* extracts.

*A. unedo* Extracts	Total Phenols Content (TPC) ^a^	Total Flavonoids Content (TFC) ^b^	Total Iridoids Content (TIC) ^c^
Leaves			
FL1	305.87 ± 1.74	178.67 ± 1.5	105.01 ± 0.75
FL2	173.33 ± 1.20	87.07 ± 1.02	102.70 ± 0.72
FL3	376.01 ± 1.93	153.62 ± 1.80	115.33 ± 0.63
FL4	298.67 ± 2.50	99.87 ± 0.62	211.31 ± 1.31
FL5	320.21 ± 3.24	137.33 ± 1.41	220.14 ± 1.02
DL1	272.67 ± 2.20	152.02 ± 1.52	118.15 ± 1.26
DL2	329.33 ± 2.32	98.01 ± 1.20	170.67 ± 2.05
DL3	290.66 ± 1.90	83.73 ± 1.11	116.22 ± 1.25
DL4	187.73 ± 1.44	99.21 ± 1.23	102.03 ± 1.23
DL5	252.12 ± 1.74	190.04 ± 1.24	135.30 ± 1.21
Fruits			
FF1	39.93 ± 0.41	26.07 ± 0.11	119.33 ± 1.30
FF2	40.06 ± 0.35	29.13 ± 0.20	104.67 ± 1.02
FF3	34.53 ± 0.44	25.13 ± 0.34	104.11 ± 1.23
FF4	35.87 ± 0.53	25.22 ± 0.23	158.67 ± 1.60
FF5	35.02 ± 0.55	26.61 ± 0.2	108.21 ± 1.24
DF1	82.20 ± 1.03	27.73 ± 0.22	147.33 ± 1.20
DF2	42.27 ± 0.21	26.02 ± 0.31	176.66 ± 1.93
DF3	81.73 ± 1.15	26.86 ± 0.20	116.61 ± 1.20
DF4	39.27 ± 0.64	27.27 ± 0.23	166.22 ± 1.01
DF5	36.13 ± 0.73	26.13 ± 0.13	195.30 ± 1.92

FL: fresh leaves; DL: dried leaves; FF: fresh fruits; DF: dried fruits. 1. Ethanolic maceration; 2. hydroalcoholic maceration; 3. decoction; 4. ethanol extraction Soxhlet apparatus; 5. ethanol ultrasound-assisted extraction. Data are reported to mean ± standard deviation (SD) (*n* = 3). ^a^ mg chlorogenic acid (CA) equivalents/g dry extract. ^b^ mg quercetin (QE) equivalents/g dry extract. ^c^ mg aucubin (AU) equivalents/g dry extract.

**Table 2:** Identification of chemical compounds in *A. unedo* leaves using the LC-ESI-QTOF-MS technique.

Compound	Rt (min)	Molecular Formula	MH^+^/ MNa^+^	Error (ppm)	Score (%)	MS fragment (*m/z*)	UV λ (nm)	Fresh Leaves					Dried Leaves					Ref.
								FL1	FL2	FL3	FL4	FL5	DL1	DL2	DL3	DL4	DL5	
*Phenolic acids*																		
Anisic acid	1.4	C_8_H_8_O_3_Na	175.0389	1.2	95		283	√	√	√	√	√	√	√	√	√	√	[42]
Caffeic acid	5.5	C_9_H_8_O_4_	181.0498	0.4	100		238, 322						√	√				[43]
Ellagic acid	12.1	C_14_H_6_O_8_	303.0136	0.8	100		255, 365	√	√	√	√	√	√	√	√	√	√	[44]
Ellagic acid 4-*O*-*β*-D-glucopyranoside	10.1	C_20_H_16_O_13_	465.0659	0.7	100	303.0136	256, 348	√	√	√	√	√	√	√	√	√	√	[46]
Ferulic acid	1.7	C_10_H_10_O_4_	195.0652	2.1	100		325	√	√	√	√	√	√	√	√	√	√	[45]
Gallic acid	3.2	C_7_H_6_O_5_	171.0287	0.3	100		217, 271	√	√	√	√	√	√	√	√	√	√	[42]
Galloyl quinic acid (3-*O*- or 5-*O*-)	3.9	C_14_H_16_O_10_	345.0814	0.3	98		Nd	√	√	√	√	√	√	√	√	√	√	[36]
Quinic acid	0.9	C_7_H_12_O_6_	193.0706	0.5	100		-	√	√	√	√	√	√	√	√	√	√	[43]
Shikimic acid gallate (3-*O*- or 5-*O*-)	7.1	C_14_H_14_O_9_	327.0711	0.7	100	174.1350	215, 277	√	√	√	√	√	√	√	√	√	√	[36]
Syringic acid	10.6	C_9_H_10_O_5_	199.0601	0.1	100		273	√	√	√	√	√	√	√	√	√	√	[44]
*Flavonoids*																		
Afzelin	14.4	C_21_H_20_O_10_	433.1131	0.1	100	287.0550	265, 301, 347	√	√	√	√	√	√	√	√	√	√	[44]
Catechin	9.4	C_15_H_14_O_6_	291.0866	1.2	100		280	√	√	√	√	√	√	√	√	√	√	[47]
Isovitexin 7-*O*-glucoside	12.9	C_27_H_30_O_15_	595.1658	0.1	100	432.3768	265, 330	√	√	√	√	√	√	√	√	√	√	[43]
Kaempferol	14.2	C_15_H_10_O_7_	287.0550	0.1	100		254, 365	√	√	√	√	√	√	√	√	√	√	[35]
Kaempferol 3-*O*-glucoside	13.2	C_21_H_20_O_11_	449.1077	0.3	100	287.0488	264, 347	√	√	√	√	√	√	√	√	√	√	[40]
Myricetin 3-*O*-xyloside	12.4	C_20_H_18_O_12_	451.0752	0.5	100	319.0435	255, 373	√	√	√	√	√	√	√	√	√	√	[47]
Naringenin 7-*O*-glucoside	12.4	C_21_H_22_O_10_	435.1257	0.2	100	273.5640	283, 332	√	√	√	√	√	√	√	√	√	√	[43]
Norbergenin	9.2	C_13_H_14_O_9_	315.0710	0.5	100		222, 289						√	√				[41]
Rutin	12.4	C_27_H_30_O_16_	611.1612	0.2	100	303.0499	253, 352	√	√	√	√	√	√	√	√	√	√	[47]
Hyperoside(*)	12.8	C_21_H_20_O_12_	465.1031	0.8	100	303.0499	254, 353	√	√	√	√	√	√	√	√	√	√	[35]
Isoquercitrin(*)	12.8	C_21_H_20_O_12_	465.1031	0.8	100	303.0499	253, 353	√	√	√	√	√	√	√	√	√	√	[35]
Myricetin	12.6	C_15_H_10_O_8_	319.0446	0.1	100		255, 375			√	√		√					[45]
Myricetin 3-*O*-rhamnopyranoside(*)	12.7	C_21_H_20_O_12_	465.1031	0.4	100	319.0389	253, 365	√	√	√	√	√	√	√	√	√	√	[39]
Quercetin 3-*O*-arabinoside(**)	13.4	C_20_H_18_O_11_	435.7749	0.3	100	303.0499	253, 353	√	√	√	√	√	√	√	√	√	√	[39]
Quercetin 3-*O*-xyloside(**)	13.5	C_20_H_18_O_11_	435.7749	0.3	100	303.0499	254, 356	√	√	√	√	√	√	√	√	√	√	[47]
Quercitrin	13.6	C_21_H_20_O_11_	449.1079	0.7	100	303.0499	254, 356	√	√	√	√	√	√	√	√	√	√	[35]
*Proanthocyanidins*																		
Gallocatechin	6.8	C_15_H_14_O_7_	307.0811	2.9	89		271			√	√		√	√	√			[47]
Epicatechin-4,6-catechin(***)	9.5	C_30_H_26_O_12_	579.1495	0.1	100	291.0851	280	√	√	√	√	√	√	√	√	√	√	[47]
Epicatechin-4,8-epicatechin(***)	11.8	C_30_H_26_O_12_	579.1492	0.3	100	291.0851	280	√	√	√	√	√	√	√	√	√	√	[47]
*Iridoids*																		
Asperuloside	1.8	C_18_H_22_O_11_	415.1214	4.4	84		239			√			√	√				[34]
Gardenoside	5.9	C_17_H_24_O_11_	405.1391	0.5	96		237	√	√	√		√	√	√	√		√	[34]
Geniposide	10.5	C_17_H_24_O_10_	389.1447	0.8	98		239						√	√				[34]
Stilbericoside	15.7	C_14_H_20_O_10_	349.1129	0.1	99		Nd								√			[48]
Unedide	1.2	C_16_H_24_O_12_	409.1340	0.4	100		Nd			√			√	√	√	√	√	[48]
Unedoside	11.2	C_14_H_20_O_10_	333.0819	0.4	95		Nd						√	√				[48]
*Phenolic glucosides*																		
Arbutin	1.8	C_12_H_16_O_7_Na	295.0793	0.8	96		230, 282						√	√				[44]
*p*-hydroxybenzoic acid glucuronide	9.8	C_13_H_14_O_9_	315.0712	0.5	94		253		√	√	√	√	√	√	√	√	√	[42]

FL: fresh leaves extracts; DL: dried leaves extracts. 1. Ethanolic maceration; 2. hydroalcoholic maceration; 3. decoction; 4. ethanol extraction Soxhlet apparatus; 5. ethanol ultrasound-assisted extraction. Rt: Retention time. √: presence. Nd: not detected. -: no UV. (*) (**)(***): Interchangeable. Ref.: references.

**Table 3:** Identification of chemical compounds in *A. unedo* fruits using the LC-ESI-QTOF-MS technique.

Compound	Rt (min)	Molecular Formula	MH^+^/ MNa^+^	Error (ppm)	Score (%)	MS Fragment (*m/z*)	UV λ (nm)	Fresh Fruits					Dried Fruits					Ref.
								FF1	FF2	FF3	FF4	FF5	DF1	DF2	DF3	DF4	DF5	
*Phenolic acids*																		
Anisic acid	1.4	C_8_H_8_O_3_Na	175.039	1.2	95		283	√	√	√	√	√	√	√	√	√	√	[42]
Ellagic acid 4-*O*-*β*-D-glucopyranoside	10.1	C_20_H_16_O_13_	465.066	0.7	100	303.0136	256, 348	√	√	√	√	√	√	√	√	√	√	[46]
Ferulic acid	1.7	C_10_H_10_O_4_	195.065	2.1	100		325	√	√	√	√	√	√	√	√	√	√	[45]
Gallic acid	3.2	C_7_H_6_O_5_	171.029	0.3	100		217, 271	√	√	√	√	√	√	√	√	√	√	[42]
Galloyl quinic acid (3- *O*- or 5-*O*-)	3.9	C_14_H_16_O_10_	345.081	0.3	98		Nd	√	√	√	√	√	√	√	√	√	√	[36]
Protocatechuic acid	6.2	C_7_H_6_O_4_	155.035	1.8	100		290	√	√	√			√					[42]
Quinic acid	0.9	C_7_H_12_O_6_	193.071	0.5	100		-	√	√	√	√	√	√	√	√	√	√	[43]
Shikimic acid gallate (3-*O*- or 5-*O*-)	7.1	C_14_H_14_O_9_	327.071	0.7	100		215, 277	√	√	√	√	√	√	√	√	√	√	[36]
Syringic acid	10.6	C_9_H_10_O_5_	199.060	0.1	100		218, 273	√	√							√		[44]
*Flavonoids*																		
Catechin	9.4	C_15_H_14_O_6_	291.087	1.2	100		280	√										[47]
Hyperoside (*)	12.8	C_21_H_20_O_12_	465.103	0.8	100	303.0499	213,278, 350						√	√	√	√	√	[35]
Isoquercitrin (*)	12.8	C_21_H_20_O_12_	465.103	0.8	100	303.0499	213,253, 353						√	√	√	√	√	[35]
Myricetin	12.6	C_15_H_10_O_8_	319.045	0.1	100		220,255, 375	√	√	√	√	√	√	√	√	√	√	[45]
Myricetin 3-*O*-rhamnopyranoside (*)	12.7	C_21_H_20_O_12_	465.103	0.4	100	319.0389	219,253, 365						√	√	√	√	√	[39]
Quercetin 3-*O*-arabinoside (**)	13.4	C_20_H_18_O_11_	435.775	0.3	100	303.0499	213,253, 353	√	√	√	√	√	√	√	√	√	√	[39]
Quercetin 3-*O*-xyloside (**)	13.5	C_20_H_18_O_11_	435.775	0.3	100	303.0499	213,254, 356	√	√	√	√	√	√	√	√	√	√	[47]
Quercitrin	13.6	C_21_H_20_O_11_	449.108	0.7	100	303.0499	213,254, 356	√	√	√	√	√	√	√	√	√	√	[35]
*Iridoids*																		
Asperuloside	1.8	C_18_H_22_O_11_	415.121	4.4	84		239									√		[34]
Stilbericoside	15.7	C_14_H_20_O_10_	349.113	0.1	99		Nd	√					√	√	√	√	√	[48]
Unedide	1.2	C_16_H_24_O_12_	409.134	0.4	100		Nd	√	√	√	√	√						[48]

FF: fresh fruits extracts, DF: dried fruits extracts. 1. Ethanolic maceration; 2. hydroalcoholic maceration; 3. decoction; 4. ethanol extraction Soxhlet apparatus; 5. ethanol ultrasound-assisted extraction. Rt: Retention time. √: presence. Nd: not detected. -: no UV. (*) (**): Interchangeable.

**Table 4:** In vitro antioxidant activity of *A. unedo* extracts.

*A. unedo*	ABTS Test IC_50_ (μg/mL)	DPPH Test IC_50_ (μg/mL)	FRAP Test IC_50_ (μM Fe (II)/g)	β-Carotene Bleaching Test IC_50_ (μg/mL)	
Leaves				30 min	60 min
FL1	6.82 ± 0.61 ****	6.89 ± 0.70 ****	94.20 ± 3.56	63.68 ± 2.06 ****	8.87 ± 0.28 ****
FL2	7.01 ± 0.72 ****	7.88 ± 0.85 ****	83.03 ± 2.50	13.19 ± 1.01 ****	32.71 ± 1.03 ****
FL3	1.16 ± 0.02	14.86 ± 1.15 ****	17.98 ± 1.77 ****	31.21 ± 1.34 ****	46.03 ± 1.04 ****
FL4	7.50 ± 0.75 ****	7.88 ± 0.64 ****	91.82 ± 3.83	27.92 ± 1.02 ****	12.82 ± 1.01 ***
FL5	8.22 ± 0.80 ****	6.89 ± 0.83 ****	84.42 ± 2.78	41.06 ± 2.04 ****	7.94 ± 0.71 ****
DL1	0.39 ± 0.03	3.94 ± 0.04 ***	17.95 ± 1.96 ****	1.85 ± 0.02	4.09 ± 0.43 ***
DL2	0.42 ± 0.04	0.98 ± 0.09	32.78 ± 2.44 ****	3.21 ± 0.03	4.28 ± 0.61 ***
DL3	1.51 ± 0.21 *	3.94 ± 0.03 ***	24.88 ± 1.96 ****	8.22 ± 0.81 ****	10.75 ± 0.66 ***
DL4	0.78 ± 0.08	27.83 ± 1.45 ****	32.49 ± 2.78 ****	13.38 ± 1.42 ****	12.63 ± 1.16 ***
DL5	1.30 ± 0.09 *	24.83 ± 1.13 ****	32.56 ± 3.92 ****	36.74 ± 1.63 ****	13.10 ± 0.83 ****
Fruits					
FF1	51.30 ± 2.55 ****	69.07 ± 2.07 ****	25.70 ± 1.12 ****	32.68% ^a^	31.77% ^a^
FF2	38.02 ± 1.30 ****	47.15 ± 1.04 ****	35.76 ± 2.13 ****	41.13% ^a^	45.78% ^a^
FF3	1.93 ± 0.54	67.19 ± 1.05 ****	24.74 ± 1.67 ****	27.08 ± 2.54 ****	28.39 ± 1.89 ****
FF4	54.62 ± 4.81 ****	56.81 ± 1.02 ****	32.08 ± 2.77 ****	186.42 ± 10.22 ****	198.44 ± 15.01 ****
FF5	50.07 ± 2.52 *****	70.94 ± 2.07 ****	20.55 ± 2.55 ****	98.76 ± 5.30 ****	46.45% ^a^
DF1	3.90 ± 1.98 **	61.93 ± 2.70 ****	24.04 ± 1.78 ****	2.54 ± 0.35 *	4.81 ± 1.23 ***
DF2	1.93 ± 0.96	53.06 ± 1.29 ****	35.31 ± 2.09 ****	19.29 ± 1.45 ****	21.92 ± 2.54 ****
DF3	2.32 ± 1.56 *	32.21 ± 2.45 ****	30.64 ± 2.08 ****	74.94 ± 3.64 ****	82.45 ± 3.64 ****
DF4	1.16 ± 0.35	49.12 ± 1.45 ****	39.59 ± 3.05 ****	25.11 ± 2.58 ****	29.52 ± 2.47 ****
DF5	4.30 ± 2.36 ***	60.94 ± 5.23 ****	24.60 ± 2.27 ****	39.09 ± 3.65 ****	48.01 ± 1.36 ****
Positive control					
Ascorbic acid	1.70 ± 0.21	5.03 ± 0.80			
BHT			63.20 ± 4.31		
Propyl gallate				1.01 ± 0.01	1.02 ± 0.01

FL: fresh leaves; DL: dried leaves; FF: fresh fruits; DF: dried fruits. 1. Ethanolic maceration; 2. Hydroalcoholic maceration; 3. Decoction; 4. Ethanol extraction Soxhlet apparatus; 5. Ethanol ultrasound-assisted extraction. BHT: butylated hydroxytoluene. ^a^ at a concentration of 100 μg/mL. Data are expressed as means ± S.D. (*n* = 3). Differences within and between groups were evaluated by one-way ANOVA followed by a multi-comparison Dunnett’s test (α = 0.05): ****p < 0.0001, ***p< 0.001, **p < 0.01, *p< 0.1 compared with the positive controls.

**Table 5:** Hypoglycaemic activity [IC_50_ (μg/mL)] of *A. unedo* extracts.

*A. unedo*	α-Amylase	α-Glucosidase
Leaves		
FL1	63.43 ± 1.68	232.73 ± 6.49 ****
FL2	191.56 ± 2.58 ****	31.38 ± 0.24
FL3	222.22 ± 3.67 ****	162.66 ± 5.47 ****
FL4	329.07 ± 4.58 ****	267.76 ± 6.36 ****
FL5	291.41 ± 10.36 ****	19.56 ± 0.22
DL1	269.51 ± 6.57 ****	379.00 ± 2.14 ****
DL2	683.80 ± 9.44 ****	146.89 ± 3.68 ****
DL3	125.87 ± 2.39 ****	125.00 ± 6.31 ****
DL4	297.54 ± 3.69 ****	193.31 ± 4.59 ****
DL5	592.71 ± 4.57 ****	201.20 ± 2.14 ****
Fruits		
FF1	20.30% ^a^	181.05 ± 9.68 ****
FF2	258.13 ± 12.36****	28.42 ± 0.82
FF3	35.14% ^a^	215.21 ± 6.57 ****
FF4	22.83% ^a^	423.77 ± 5.34 ****
FF5	27.18% ^a^	40.25 ± 0.79
DF1	107.51 ± 9.15 ****	255.50 ± 7.89 ****
DF2	146.51 ± 8.98 ****	316.81 ± 9.68 ****
DF3	295.14 ± 3.02 ****	456.23 ± 2.56 ****
DF4	77.51 ± 1.08 **	151.27 ± 4.63 ****
DF5	120.58 ± 3.48 ****	239.73 ± 6.58 ****
Positive control		
Acarbose	50.01 ± 1.43	35.50 ± 1.10

FL: fresh leaves; DL: dried leaves; FF: fresh fruits; DF: dried fruits. 1. Ethanolic maceration; 2. hydroalcoholic maceration; 3. decoction; 4. ethanol extraction Soxhlet apparatus; 5. ethanol ultrasound-assisted extraction. ^a^ at a concentration of 1 mg/mL. Data are expressed as means ± S.D. (*n* = 3). Differences within and between groups were evaluated by one-way ANOVA followed by a multi-comparison Dunnett’s test (α = 0.05): ****p < 0.0001, ***p < 0.001, **p < 0.01, *p < 0.1 compared with the positive controls.

## References

[1] Islam M.A., Alam F., Solayman M., Khalil M.I., Kamal M.A., Gan S.H. (2016). Dietary phytochemicals: Natural swords combating inflammation and oxidation-mediated degenerative diseases. Oxidative Med. Cell. Longev..

[2] Fischer R., Maier O. (2015). Interrelation of oxidative stress and inflammation in neurodegenerative disease: Role of TNF. Oxidative Med. Cell. Longev..

[3] Gasparetto J.C., Martins C.A., Hayashi S.S., Otuky M.F., Pontarolo R. (2012). Ethnobotanical and scientific aspects of *Malva sylvestris* L.: A millennial herbal medicine. J. Pharm. Pharmacol..

[4] Abidi E., Habib J., Mahjoub T., Belhadj F., Garra M., Elkak A. (2016). Chemical composition, antioxidant and antibacterial activities of extracts obtained from the roots bark of *Arbutus andrachne* L. a Lebanese tree. Int. J. Phytomed..

[5] Watzl B. (2008). Anti-inflammatory effects of plant-based foods and of their constituents. Int. J. Vitam. Nutr. Res..

[6] Acquaviva R., Iauk L. (2010). Natural polyphenols as anti-inflammatory agents. Front. Biosci..

[7] Ahh Y.G., Shin J.H., Kim H.Y., Khim J., Lee M.K., Hong J. (2007). Application of solid phase extraction coupled with freezing lipid filtration clean-up for the determination of the determination of endocrine-disrupting phenols in fish. Anal. Chim..

[8] Co M., Fagerlund A., Engman L., Sunnerheim K., Sjoberg P.J.R., Turner C. (2012). Extraction of antioxidants from Spruce (*Picea abies*) bark using ecofriendly solvents. Phytochem. Anal..

[9] Hayouni E.A., Abedrabba M., Bouix M., Hamdi M. (2007). The effects of solvents and extraction method on the phenolic contents and biological activities in vitro of Tunisian *Quercus coccifera* L. and *Juniperus phoenica* L. fruit extracts. Food Chem..

[10] Ollanketo M., Peltoketo A., Hartonen K., Hiltunen R., Riekkola M.L. (2002). Extraction of sage (*Salvia officinalis* L.) by pressurized hot water and conventional methods: Activity of the extracts. Eur. Food Res. Technol..

[11] Morgado S., Morgado M., Plácido A.I., Roque F., Duarte A.P. (2018). *Arbutus unedo* L.: From traditional medicine to potential uses in modern pharmacotherapy. J. Ethnopharmacol..

[12] Maldini M., D’Urso G., Pagliuca G., Petretto G.L., Foddai M., Gallo F.R., Multari G., Caruso D., Montoro P., Pintore G. (2019). HPTLC-PCA complementary to HRMS-PCA in the case study of *Arbutus unedo* antioxidant phenolic profiling. Foods.

[13] Tenuta M.C., Tundis R., Xiao J., Loizzo M.R., Dugay A., Deguin B. (2018). *Arbutus* species (Ericaceae) as source of valuable bioactive products. Crit. Rev. Food Sci. Nutr..

[14] Mrabti H.N., El Abbes Faouzi M., Mayuk F.M., Makrane H., Limas-Nzouzi N., Dibong S.D., Cherrah Y., Elombo F.K., Gressier B., Desjeux J.F. (2019). *Arbutus unedo* L., (Ericaceae) inhibits intestinal glucose absorption and improves glucose tolerance in rodents. J. Ethnopharmacol..

[15] Oliveira I., Coelho V., Baltasar R., Pereira J.A., Baptista P. (2009). Scavenging capacity of strawberry tree (*Arbutus unedo* L.) leaves on free radicals. Food Chem. Toxicol..

[16] Orak H.H., Yagar H., Isbilir S.S., Demirci A.Ş., Gümüş T., Ekinci N. (2011). Evaluation of antioxidant and antimicrobial potential of strawberry tree (*Arbutus unedo* L.) leaf. Food Sci. Biotechnol..

[17] Orak H.H., Aktas T., Yagar H., Isbilir S.S., Ekinci N., Sahin F.H. (2012). Effects of hot air and freeze-drying methods on antioxidant activity, colour and some nutritional characteristics of strawberry tree (*Arbutus unedo* L.) fruit. Food Sci. Technol. Int..

[18] Benayad Z., Martinez-Villaluenga C., Frias J., Gomez-Cordoves C., Es-Safi N.E. (2014). Phenolic composition, antioxidant and anti-inflammatory activities of extracts from Moroccan Opuntia ficus-indica flowers obtained by different extraction methods. Ind. Crops Prod..

[19] Pintać D., Majkić T., Torović L., Orčić D., Beara I., Simin N., Mimica–Dukić N., Lesjak M. (2018). Solvent selection for efficient extraction of bioactive compounds from grape pomace. Ind. Crops Prod..

[20] Gao X., Ohlander M., Jeppsson N., Björk L., Trajkovski V. (2000). Changes in antioxidant effects and their relationship to phytonutrients in fruits of Sea buckthorn (*Hippophae rhamnoides* L.) during maturation. J. Agric. Food Chem..

[21] Yoo K.M., Lee C.H., Lee H., Moon B.K., Lee C.Y. (2008). Relative antioxidant and cytoprotective activities of common herbs. Food Chem..

[22] Lin Y., Xu W., Huang M., Li H., Ye M., Zhang X., Chu K. (2015). Qualitative and quantitative analysis of phenolic acids, flavonoids and iridoid glycosides in Yinhua Kanggan tablet by UPLC-QqQ-MS/MS. Molecules.

[23] Loizzo M.R., Tundis R., Chandrika U.G., Abeysekera A.M., Menichini F., Frega N.G. (2010). Antioxidant and antibacterial activities on foodborne pathogens of *Artocarpus heterophyllus* Lam. (Moraceae) leaves extracts. J. Food Sci..

[24] Amin I., Zamaliah M.M., Chin W.F. (2004). Total antioxidant activity and phenolic content in selected vegetables. Food Chem..

[25] Leeuw R.W., Kevers C., Pincemail J., Defraigne J.O., Dommes J. (2014). Antioxidant capacity and phenolic composition of red wines from various grape varieties: Specificity of Pinot Noir. J. Food Compos. Anal..

[26] Sun T., Tanumihardjo S.A. (2007). An integrated approach to evaluate food antioxidant capacity. J. Food Sci..

[27] Malfa G.A., Tomasello B., Sinatra F., Villaggio G., Amenta F., Avola R., Renis M. (2014). “Reactive” response evaluation of primary human astrocytes after methylmercury exposure. J. Neurosci. Res..

[28] Sharma J.N., Al-Omran A., Parvathy S.S. (2007). Role of nitric oxide in inflammatory diseases. Inflammopharmacology.

[29] Saijo F., Milsom A.B., Bryan N.S., Bauer S.M., Vowinkel T., Ivanovic M., Andry C., Granger D.N., Rodriguez J., Feelisch M. (2010). On the dynamics of nitrite, nitrate and other biomarkers of nitric oxide production in inflammatory bowel disease. Nitric Oxide.

[30] Tundis R., Bonesi M., Sicari V., Pellicanò T.M., Tenuta M.C., Leporini M., Menichini F., Loizzo M.R. (2016). *Poncirus trifoliata* (L.) Raf.: Chemical composition, antioxidant properties and hypoglycaemic activity via the inhibition of α-amylase and α-glucosidase enzymes. J. Funct. Foods.

[31] Isbilir S.S., Orak H.H., Yagar H., Ekinci N. (2012). Determination of antioxidant activities of strawberry tree (*Arbutus unedo* L.) flowers and fruits at different ripening stages. Acta Sci. Pol. Technol..

[32] Bouzid K., Toumi F.B. (2014). Geoclimatic influences on the constituents and antioxidant activity of extracts from the fruit of *Arbutus unedo* L.. Phytothérapie.

[33] Salem I.B., Ouesleti S., Mabrouk Y., Landolsi A., Saidi M., Boulilla A. (2018). Exploring the nutraceutical potential and biological activities of *Arbutus unedo* L. (Ericaceae) fruits. Ind. Crops Prod..

[34] Karikas G.A. (1993). Iridoids from *Arbutus unedo*. Fitoterapia.

[35] Maleš Ž., Plazibat M., Vundać V.B., Žuntar I. (2006). Qualitative and quantitative analysis of flavonoids of the strawberry tree—*Arbutus unedo* L. (*Ericaceae*). Acta Pharm..

[36] Pawlowska A.M., De Leo M., Braca A. (2006). Phenolics of *Arbutus unedo* L. (Ericaceae) fruits:  identification of anthocyanins and gallic acid derivatives. J. Agric. Food Chem..

[37] Alarcão-E-Silva M.L.C.M.M., Leitão A.E.B., Azinheira H.G., Leitão M.C.A. (2001). The arbutus berry: Studies on its color and chemical characteristics at two mature stages. J. Food Compos. Anal..

[38] Mendes L., de Freitas V., Baptista P., Carvalho P. (2011). Comparative antihemolytic and radical scavenging activities of strawberry tree (*Arbutus unedo* L.) leaf and fruit. Food Chem. Toxicol..

[39] Sakar M.K., Berkman M.Z., Nahrstedt A., Albrecht M. (1992). Flavonoids of *Arbutus Andrachne* L. leaves. J. Pharm..

[40] Su Z. (2012). Anthocyanins and flavonoids of *Vaccinium* L.. Pharm. Crops.

[41] Taneyama M., Yoshida S., Kobayashi M., Hasegawa M. (1983). Isolation of norbergenin from *Saxifraga stolonifera*. Phytochemistry.

[42] Ayaz F.A., Kucukislamoglu M., Reunanen M. (2000). Sugar, non-volatile and phenolic acids composition of strawberry tree (*Arbutus unedo* L. var. *ellipsoidea*) fruits. J. Food Compos. Anal..

[43] El Shibani F.A.E.S. (2017). A Pharmacognostical Study of *Arbutus pavarii* Pampan. Family *Ericaceae* and *Sarcopoterium spinosum* L. Family Rosaceae Growing in Libya. Ph.D. Thesis.

[44] Guendouze-Bouchefa N., Madani K., Chibane M., Boulekbache-Makhlouf L., Hauchard D., Kiendrebeogo M., Stévigny C., Okusa P.N., Duez P. (2015). Phenolic compounds, antioxidant and antibacterial activities of three Ericaceae from Algeria. Ind. Crops Prod..

[45] Hamad H.H., Mariam I.H.H., Gonaid H., Mojahidul I. (2011). Comparative phytochemical and antimicrobial investigation of some plants growing in Al Jabal Al-Akhdar. J. Nat. Prod. Plant Resour..

[46] Yoshida T., Amakura Y., Liu Y.Z., Okuda T. (1994). Tannins and related polyphenols of euphorbiaceous plants. XI. Three new hydrolyzable tannins and a polyphenol glucoside from Euphorbia humifusa. Chem. Pharm. Bull..

[47] Pallauf K., Rivas-Gonzalo J.C., del Castillo M.D., Cano M.P., de Pascual-Teresa S. (2008). Characterization of the antioxidant composition of strawberry tree (*Arbutus unedo* L.) fruits. J. Food Compos. Anal..

[48] Davini E., Davini P., Esposito C., Iavarone C.T. (1981). Structure and configuration of unedide, an iridoid glucoside from *Arbutus unedo*. Phytochemistry.

[49] Pellegrini M., Serafini B., Colombi D., del Rio S., Salvatora M., Bianchi Brighenti F. (2003). Total antioxidant capacity of plant foods, beverages and oils consumed in Italy by three different in vitro assays. J. Nutr..

[50] Takahashi H., Kosaka M., Watanabe Y., Nakade K., Fukuyama Y. (2003). Synthesis and neuroprotective activity of bergenin derivatives with antioxidant activity. Bioorg. Med. Chem..

[51] Tangmouo J.G., Ho R., Lannang M.A., Komguem J., Lontsi A.T., Lontsi D., Hostettmann K. (2009). Norbergenin derivatives from the stem bark of *Diospyros sanza-minika* (Ebenaceae) and their radical scavenging activity. Phytochem. Lett..

[52] Zamarrud, Ali I., Hussain H., Ahmad V.U., Qaiser M., Amyn A., Mohammad F.V. (2011). Two new antioxidant bergenin derivatives from the stem of *Rivea hypocrateriformis*. Fitoterapia.

[53] Chen J.H., Ho C.T. (1997). Antioxidant activities of caffeic acid and its related hydroxycinnamic acid compounds. J. Agric. Food Chem..

[54] Gülçin İ. (2006). Antioxidant activity of caffeic acid (3,4-dihydroxycinnamic acid). Toxicology.

[55] Masek A., Chrzescijanska E., Latos M. (2016). Determination of antioxidant activity of caffeic acid and *p*-coumaric acid by using electrochemical and spectrophotometric assays. Int. J. Electrochem. Sci..

[56] Sidoryk K., Jaromin A., Filipczak N., Cmoch P., Cybulski M. (2018). Synthesis and antioxidant activity of caffeic acid derivatives. Molecules.

[57] Bang S.H., Han S.J., Kim D.H. (2008). Hydrolysis of arbutin to hydroquinone by human skin bacteria and its effect on antioxidant activity. J. Cosmet. Dermatol..

[58] Takebayashi J., Ishii R., Chen J., Matsumoto T., Ishimi Y., Tai A. (2010). Reassessment of antioxidant activity of arbutin: Multifaceted evaluation using five antioxidant assay systems. Free Radic. Res..

[59] Magnani C., Isaac V.L.B., Correa M.A., Salgado H.R.N. (2014). Caffeic acid: A review of its potential use in medications and cosmetics. Anal. Methods.

[60] Gálvez M., Martin-Cordero C., Houghton P.J., Ayuso M.J. (2005). Antioxidant activity of *Plantago bellardii* All. Phytother. Res..

[61] Mariotto S., Ciampa A.R., Carcereri de Prati A., Darra E., Vincenzi S., Sega M., Cavalieri E., Shoji K., Suzuki H. (2008). Aqueous extract of *Arbutus unedo* Inhibits STAT1 activation in human breast cancer cell line MDA-MB-231 and human fibroblasts through SHP2 activation. Med. Chem..

[62] Bonesi M., Loizzo M.R., Acquaviva R., Malfa G.A., Aiello F., Tundis R. (2017). Anti-inflammatory and antioxidant agents from *Salvia* genus (Lamiaceae): An assessment of the current state of knowledge. Antiinflamm. Antiallergy Agents Med. Chem..

[63] Robertson R.P., Harmon J., Tran P.O., Tanaka Y., Takahashi H. (2003). Glucose toxicity in beta-cells: Type 2 diabetes, good radicals gone bad, and the glutathione connection. Diabetes.

[64] Halban P.A., Polonsky K.S., Bowden D.W., Hawkins M.A., Ling C., Mather K.J., Powers A.C., Rhodes C.J., Sussel L., Weir G.C. (2014). β-cell failure in type 2 diabetes: Postulated mechanisms and prospects for prevention and treatment. Diabetes Care.

[65] Bnouham M., Merhfour F.Z., Ziyyat A., Aziz M., Legssyer A., Mekhfi H. (2010). Antidiabetic effect of some medicinal plants of Oriental Morocco in neonatal non-insulin-dependent diabetes mellitus rats. Hum. Exp. Toxicol..

[66] Mrabti H.N., Sayah K., Jaradat N., Kichou F., Ed-Dra A., Belarj B., Cherrah Y., Faouzi M.E.A. (2018). Antidiabetic and protective effects of the aqueous extract of *Arbutus unedo* L. in streptozotocin-nicotinamide-induced diabetic mice. J. Complement. Integr. Med..

